# Predicting response of immunotherapy and targeted therapy and prognosis characteristics for renal clear cell carcinoma based on m1A methylation regulators

**DOI:** 10.1038/s41598-023-39935-4

**Published:** 2023-08-04

**Authors:** Lei Li, Hongwei Tan, Jiexue Zhou, Fengming Hu

**Affiliations:** 1 Department of Urology, Union Hospital, Tongji Medical College, Huazhong University of Science and Technology, Wuhan, Hubei People’s Republic of China; 2 Department of Organ Transplantation, Guangdong Second Provincial General Hospital, Guangzhou, Guangdong People’s Republic of China

**Keywords:** Cancer, Immunology, Biomarkers, Oncology, Urology

## Abstract

In recent years, RNA methylation modification has been found to be related to a variety of tumor mechanisms, such as rectal cancer. Clear cell renal cell carcinoma (ccRCC) is most common in renal cell carcinoma. In this study, we get the RNA profiles of ccRCC patients from ArrayExpress and TCGA databases. The prognosis model of ccRCC was developed by the least absolute shrinkage and selection operator (LASSO) regression analysis, and the samples were stratified into low–high risk groups. In addition, our prognostic model was validated through the receiver operating characteristic curve (ROC). “pRRophetic” package screened five potential small molecule drugs. Protein interaction networks explore tumor driving factors and drug targeting factors. Finally, polymerase chain reaction (PCR) was used to verify the expression of the model in the ccRCC cell line. The mRNA matrix in ArrayExpress and TCGA databases was used to establish a prognostic model for ccRCC through LASSO regression analysis. Kaplan Meier analysis showed that the overall survival rate (OS) of the high-risk group was poor. ROC verifies the reliability of our model. Functional enrichment analysis showed that there was a obviously difference in immune status between the high-low risk groups. “pRRophetic” package screened five potential small molecule drugs (A.443654, A.770041, ABT.888, AG.014699, AMG.706). Protein interaction network shows that epidermal growth factor receptor [EGRF] and estrogen receptor 1 [ESR1] are tumor drivers and drug targeting factors. To further analyze the differential expression and pathway correlation of the prognosis risk model species. Finally, polymerase chain reaction (PCR) showed the expression of YTHN6-Methyladenosine RNA Binding Protein 1[YTHDF1], TRNA Methyltransferase 61B [TRMT61B], TRNA Methyltransferase 10C [TRMT10C] and AlkB Homolog 1[ALKBH1] in ccRCC cell lines. To sum up, the prognosis risk model we created not only has good predictive value, but also can provide guidance for accurately predicting the prognosis of ccRCC.

## Introduction

Clear cell renal cell carcinoma (ccRCC) is the most common subtype, and the degree of malignancy and prognosis are the worst. According to relevant statistics, in 2015, the number of deaths from kidney cancer in my country reached 23,400 in China^1^. The main treatments for ccRCC currently include surgery, radiotherapy, and chemotherapy. However, high recurrence and metastasis rates still affect patient outcomes^2,3^. Tumor immune infiltration was a significant role in the occurrence and development of tumors which was demonstrated recently^4^. Among them, it is pointed out in the Japanese clinical treatment plan that pembrolizumab combined with lenvatinib can effectively improve the prognosis of metastatic renal cell carcinoma^5^. Many evidences indicate that RNA chemical modification plays a key role in basic biology processes such as protein production, cell differentiation, cell signal transmission and it has the potential to become a therapeutic target in diseases^6–9^. N1- methyladenosine (m1A) is widely distributed in various RNA and mitochondrial transcripts^10^. It has been confirmed that the imbalance of m1A affects a variety of biology processes, including cell proliferation and cell death, which are mainly related to tumors^11,12^. The m1A adjustment factors are divided into “writers”- (TRMT10C, TRMT61B, TRMT6, and TRMT61A), “readers”- (YTHDF1, YTHDF2, YTHDF3 and YTHDC1) and “easers”- (ALKBH1 and ALKBH3)^10,13,14^. Recent studies have found the level of gene expression regulated by m1A is related to tumor progression. In gastric cancer, m1A downstream gene is related to cell proliferation, and m1A gene is reliably related to mTOR^15^. The role of m1A regulatory gene in the progression of ccRCC has not been reported in the literature. The development of immunocheckpoint inhibitors (ICIs) has changed the treatment of advanced renal cell carcinoma (RCC), but most RCC patients still cannot obtain lasting clinical benefits^16^. Exploring the immune infiltration and immune checkpoint was vital for improving the curative effect of ccRCC. Therefore, in this study, data from the Cancer Genome Map (TCGA) database and the ArrayExpress database were used to clarify the biological function and network of m1A regulatory gene^17^, and determine the role of m1A regulatory gene in the prognosis of ccRCC. The risk model and nomograph of m1A regulatory gene were constructed to determine the prognosis of ccRCC. The relationship between risk score, immune checkpoint and immune infiltration was also discussed. Finally, using “pRRophetic” package potential small molecule targeted drugs were screened through high-risk groups. Therefore, this study focuses on exploring the impact of m1A methylation regulator on ccRCC, providing insight into anti-tumor immune response and screening small molecule drugs targeting risk label, which will help to develop more effective immunotherapy strategies.

## Materials and methods

### Data collection and processing

This study contains two databases. From the Cancer Genome Atlas (https://portal.gdc.cancer.gov/), RNA expression matrix and clinical data (FPKM) were downloaded, including 611 samples (72 normal samples and 539 tumor samples). ArrayExpress (https://www.ebi.ac.uk/arrayexpress) database includes 101 tumor samples. The “sva” package in R is combined with microarray data to eliminate the batch effect of different platforms.

### Expression differences and survive in N1-methyladenosine-related regulatory factors

We included 10 m1A methylation regulating genes (YTH N6-Methyladenosine RNA Binding Protein 1[*YTHDF1*], TRNA Methyltransferase 61B [*TRMT61B*], TRNA Methyltransferase 10C [*TRMT10C*], AlkB Homolog 1[*ALKBH1*], TRNA Methyltransferase 6[*TRMT6*], TRNA Methyltransferase 61A [*TRMT61A*], YTH N6-Methyladenosine RNA Binding Protein 2[*YTHDF2*], YTH N6-Methyladenosine RNA Binding Protein 3[*YTHDF3*], YTH Domain Containing 1[*YTHDC1*], and AlkB Homolog 3[*ALKBH3*]). The genes expression was compared in tumor group and normal tissue group. ‘Survival’ package is used to get the OS of the gene expression.

### Prognostic characteristics of N1-methyladenosine related regulators

We use the ‘sva’ package to eliminate the batch effect and merge TCGA and ArrayExpress database samples^18^. Based on TCGA, we randomly divided 530 ccRCC samples into two groups using a 1:1 ratio (TCGA-V1 and TCGA-V2). Defining TCGA-V1and ArrayExpress as training groups (n = 366) and TCGA-V2 as validation groups (n = 265) (Deleted the stage column with more clinical information missing). The risk score was calculated as follows:

$$Risk\,\,score\, = \,(Exp_{gene1} \, \times \,Coef_{gene1} )\, + \,(Exp_{gene2} \, \times \,Coef_{gene2} )\, + \, \ldots \ldots \, + \,(Exp_{gene(n)} \, \times \,Coef_{gene(n)} ).$$

According the median risk score, high- and low-risk groups were divided. Next, independent prognostic risk factors were determined by univariate and multivariate Cox regression analyses. The difference of overall survival rate (OS) between high-risk group and low-risk group was analyzed to Kaplan Meier method. Finally, the effectiveness of the risk model was verified in the training and validation groups by receiver operating characteristic (ROC) curves.

### Construction of risk signature and nomogram model

We combine Lasso’s results with clinical characteristics to obtain the heat map using the ‘pheatmap’ package. In the multivariate Cox regression analysis, we take p < 0.001 as standard to construct the prognostic nomogram model. The model’s performance was predicted to the calibration curve and C-index. The model’s predictive ability was verified by 1-, 3-, and 5-year ROC curves. And the OS of high-risk groups was displayed by survival curve.

### Evaluation of immune infiltration and immune infiltration microenvironment

Abundance of tumor immune microenvironment (TME) infiltrating cells was calculated by single sample gene set enrichment analysis (ssGSEA) algorithm^19^. The Stromal score, ESTIMATE score, and immune score in a single sample were assessed by ESTIMATE^20,21^. Next, the difference of immune cell infiltration between two groups were analyzed by CIBERSORT algorithm^22^.

### Gene set variation analysis

Gene set variation analysis (GSVA) was performed using the “GSVA” package in R to clarify the differences in biological processes based on N1-methyladenosine^19^. From the MSigDB database (http://www.gsea-msigdb.org/gsea/msigdb), the “c2.cp.kegg. v7.4. symbols” gene sets were obtained.

### Gene ontology (GO) and KEGG pathway enrichment analyses

The ‘ggplot2’ package was analyzed to GO and KEGG pathway enrichment of the DEGs. The cutoff value was considered meaningful as p < 0.01 and q < 0.05.

### Comprehensive riskscore screening potential drugs for ccRCC

We used the “pRRophetic” package to analyze the potential therapeutic drugs that may be related to clear cell renal cell carcinoma in low–high risk groups^23^. The results are represented by box diagram. PubChem (https://pubchem.ncbi.nlm.nih.gov/) was used to obtain the molecular structure of compounds^24^.

### Construction of PPI network and correlation analysis

Protein Interaction Network Analysis (PINA) platform is a management platform for building protein interaction networks (https://omics.bjcancer.org/pina/)^25^. We screened binding genes based on four independent prognostic genes, and identified cancer drivers and drug targeting factors in the interaction network. Interacted genes using a web tool (http://bioinformatics.psb.ugent.be/webtools/Venns/). (The correlation coefficient > 0.1). The “GSVA” package is used to obtain the correlation between genes and pathways (method = “sgsea”).

### Cell culture and signature gene expression

Human ccRCC (769-P) and immortalized proximal convoluted tubule epithelial (HK2) cell lines were purchased from the Chinese Academy of Sciences cell bank. They were cultured with 1640 medium (Nanjing Kaigen Biotechnology Co., Ltd., China) and Durbek Modified Eagle Medium (DMEM) (Kaigen Biotechnology Co., Ltd.) respectively. Culture conditions: the culture medium contains 10% fetal bovine serum at 37 °C and 5% carbon dioxide respectively (Shanghai Bioindustry Corporation, China). Use Takara’s kit to extract RNA to detect the difference of mRNA expression. GAPDH as internal reference. The primers were as follows: TRMT10C: Forward, TCTCTGCACTCCTGGGGTTT, Reverse, TGGCACCAAAAACCTGGTGAA. TRMT61B: Forward, CAGGCTCTGGTGGAATGAGC, Reverse, ACATATCCAAAGCTACTGCGTCAA. ALKBH1: Forward, TTGCTGTCATTCAGCTTTGGAC, Reverse, CCTCTAGGCAGTGAGGCAGG. Differences in tissue protein expression originate from The Human Protein Atlas database.

### Cell transfection and apoptosis of two genes

Overexpression (OE) of EGFR and ESR1 in 769-P cell line to prove the influence of the two genes on ccRCC apoptosis. The overexpressing adeno-associated virus-9 of EGFR and ESR1 was purchased from Gima Genomics. 769-P was transfected by recombinant adenovirus. Groups were set as transfection negative control (NC) group and transfection OE group. According to the manufacturer's instructions, the apoptosis was detected by Annexin VAPC and PI (KeyGEN BioTECH) staining, and using flow cytometry (FACSCALIBUR, Becton Dickinson, USA).

### Statistical analyses

All analyses were based on R (version 3.6.1). The “reshape2” and “ggpubr” packages were used to visualize differential gene expression. The “glmnet” package was used to screen prognostic factors. The “survivalROC” and “survminer” package was generated to ROC curves and survival curve. The C-index and calibration curves were calculated to “rms” package. P < 0.05 were considered statistically significant.

## Results

### Expression pattern of the N1-methyladenosine regulatory factors in ccRCC

**Figure 1:**
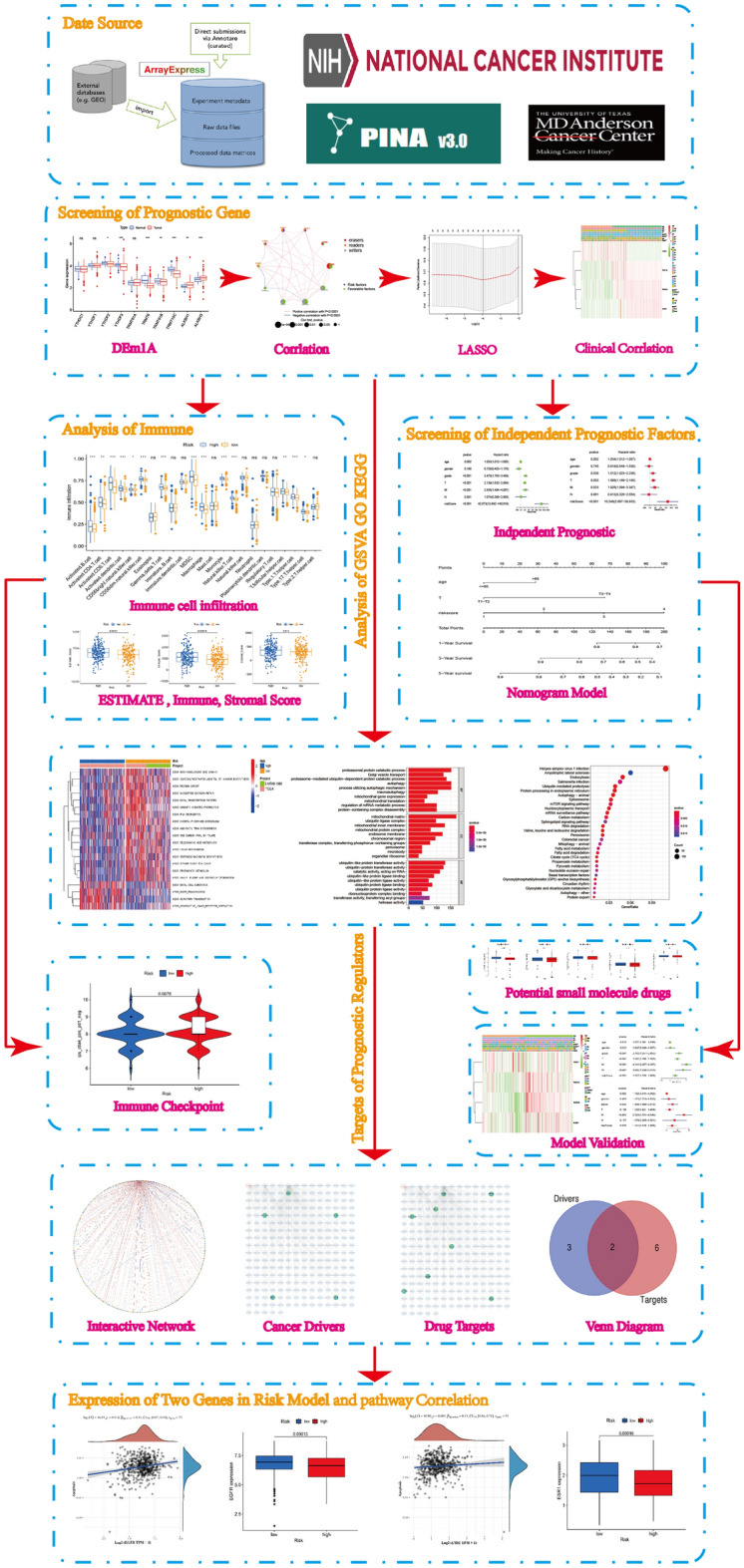
The work flow chart.

**Figure 2:**
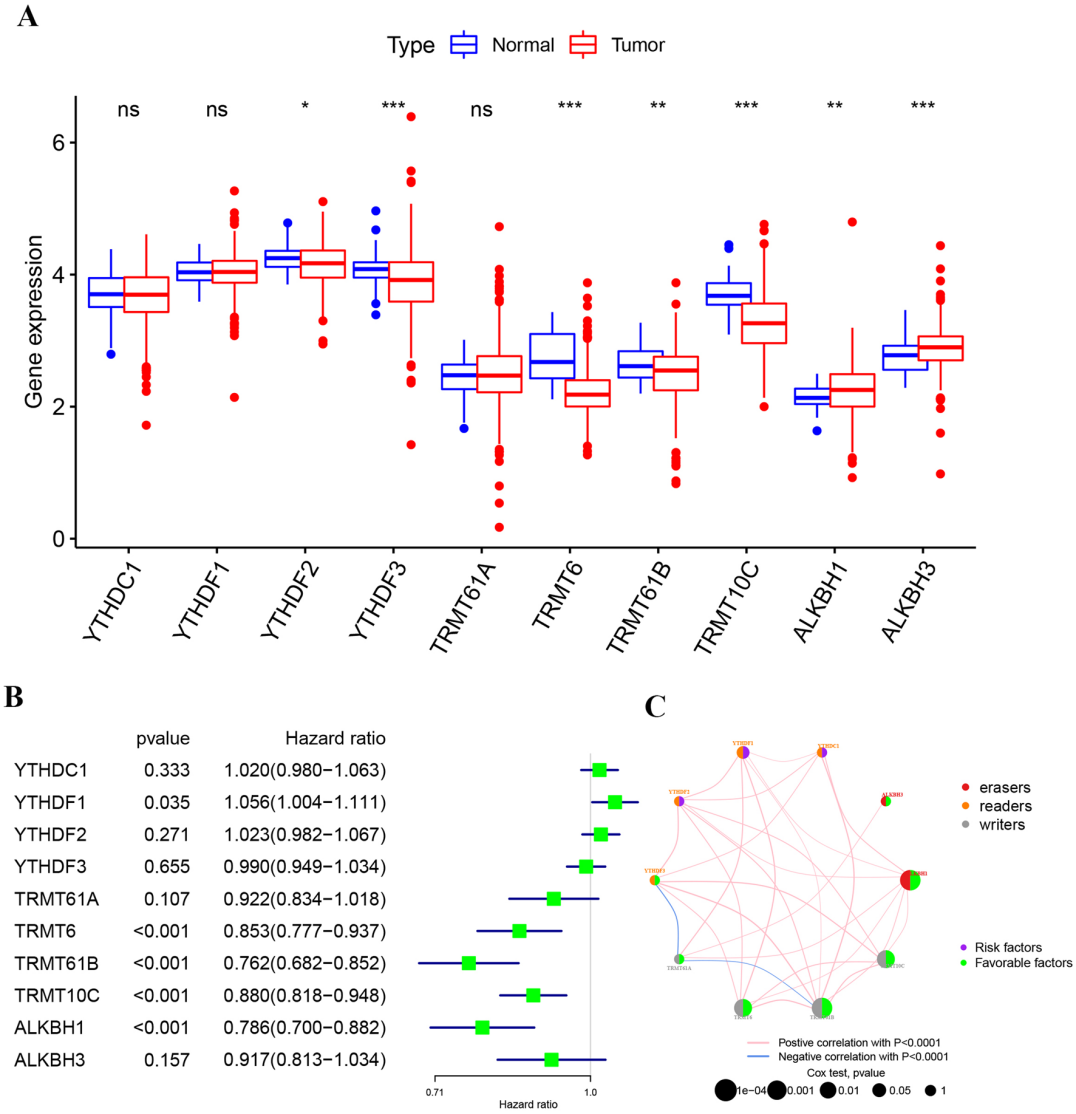
Prognostic relevance of m1A regulators in clear cell renal cell carcinoma. (**A**) The expression of 10 m1A methylation regulators between tumor tissues and normal controls (***p < 0.001). (**B**) The prognostic analyses for 10 m1A regulators using univariate Cox regression model. (**C**) The interaction between m1A regulators in clear cell renal cell carcinoma. The left part of the circle represents the category of each regulator. Erasers, red; readers, orange; writers, gray. The right part of the circle represents the survival impact of each regulator. Favorable factors of prognosis, green; risk factors of prognosis, purple (*N* node metastasis, *T* tumor, *M* metastasis).

The workflow of the study is shown in Fig. 1. Table 1 lists the m1A regulator genes. The expression pattern of 10 regulatory genes in ccRCC is shown (Fig. 2A). YTHDF2, YTHDF3, TRMT6, TRMT61B, and TRMT10C were low expression in ccRCC. ALKBH1 and ALKBH3 were high expression in ccRCC. The interaction of m1A regulator and its predicted values in ccRCC are shown (Fig. 2B,C). Our results indicate that YTHDF1, TRMT6, TRMT61B, TRMT10C and ALKBH1 affect prognosis. Where, YTHDF1 was risk factor and TRMT6, TRMT61B, TRMT10C and ALKBH1 were favorable factors.

**Table 1:** Classification of m1A regulatory factors.

Regulators	Type
TRMT10C	Writer
TRMT61B	Writer
TRMT6	Writer
TRMT61A	Writer
YTHDF1	Reader
YTHDF2	Reader
YTHDF3	Reader
YTHDC1	Reader
ALKBH1	Easer
ALKBH3	Easer

### Prognostic model of m1A regulatory factors in ccRCC

**Figure 3:**
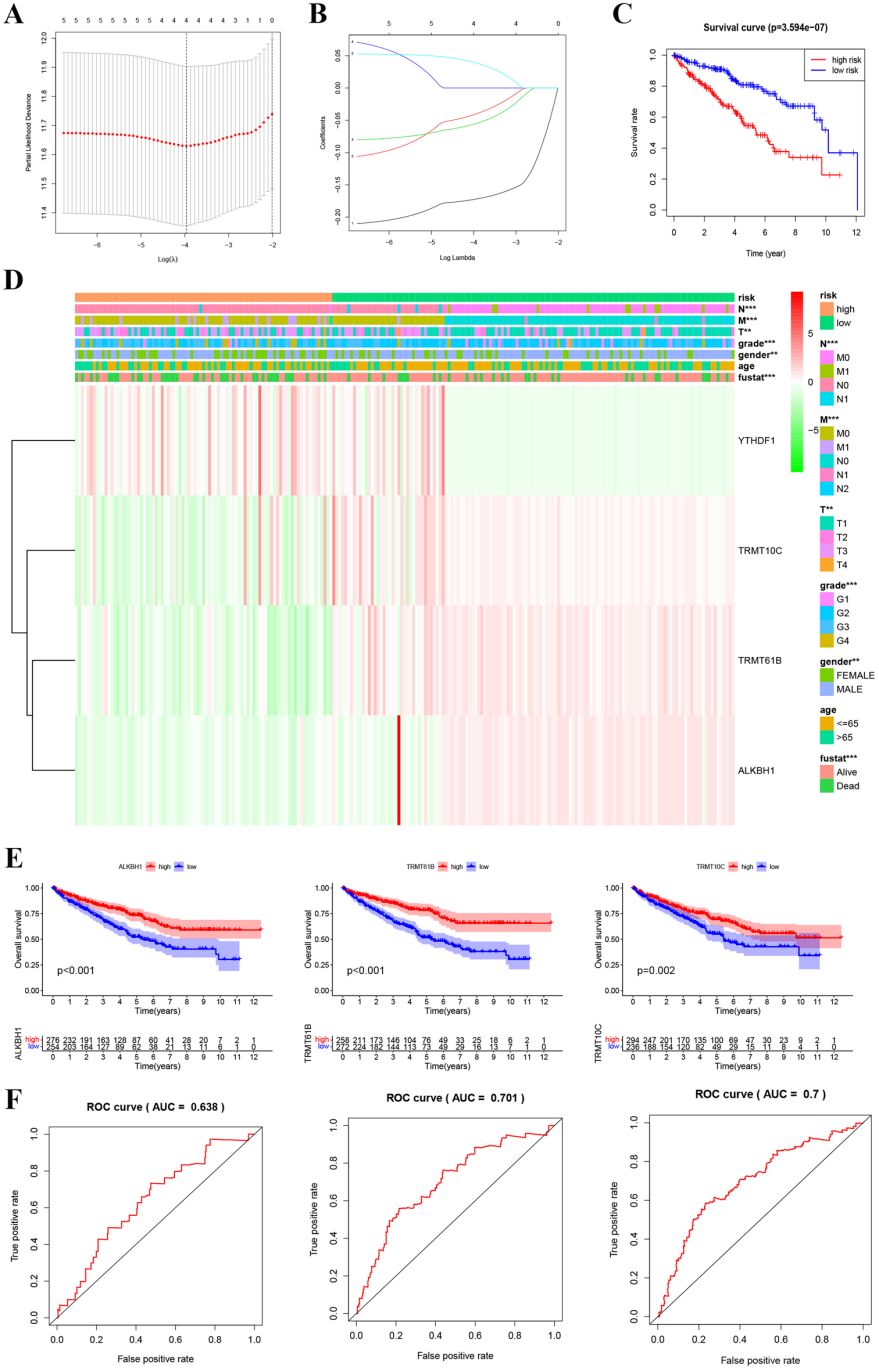
Construction of the risk signature according to the m1A RNA methylation regulators. (**A**,**B**) LASSO coefficient profiles of the five regulators. Cross validation for tuning parameter selection in the LASSO model. (**C**) The K–M analysis showed that patients in the low-risk group presented better OS than those in the high-risk group. This analysis was based on the survival information of samples in the training set. The red line represents the high-risk cluster, whereas the blue line indicates the low-risk cluster. (**D**) The training set of the heat map of the relationship between the gene expression of the corresponding four regulatory factors and clinical features. *p < 0.05, **p < 0.01, and ***p < 0.001. (**E**) Kaplan–Meier survival curves for OS of four regulatory factors. (**F**) The training set of the ROC curve for evaluating the prediction efficiency of the prognostic signature.

We chose YTHDF1, TRMT6, TRMT61B, TRMT10C and ALKBH1 carrying out Lasso regression analysis. The results showed that four regulatory genes (YTHDF1, TRMT61B, TRMT10C and ALKBH1) were identified as prognostic factors (Fig. 3A,B; Table S1). The protective factors include TRMT61B, TRMT10C and ALKBH1 (hazard ratio [HR] < 1). Risk factor was YTHDF1 (HR > 1). Next, a significantly shorter OS in high-risk group than of those in low-risk group (Fig. 3C). In addition, the relationship between the four prognostic factors and various clinical features and risks is shown by heat maps in training group. The risk correlated with node (N), tumor (T), metastasis (M), gender, fustat, and grade. In high-risk group, YTHDF1 was highly expressed, and in the low-risk group, TRMT61B, TRMT10C and ALKBH1 were highly expressed (Fig. 3D). High TRMT61B, TRMT10C and ALKBH1 expression corresponds to a good prognosis (Fig. 3E). Unfortunately, the expression of YTHDF1 is meaningless to OS (Table S2). 0.638, 0.701 and 0.7 are the areas under the 1-year, 3-year and 5-year ROC curves (AUC) (Fig. 3F). These results showed our prediction model was applicative.

### Independent prognostic factor and Norman graph model construction

**Figure 4:**
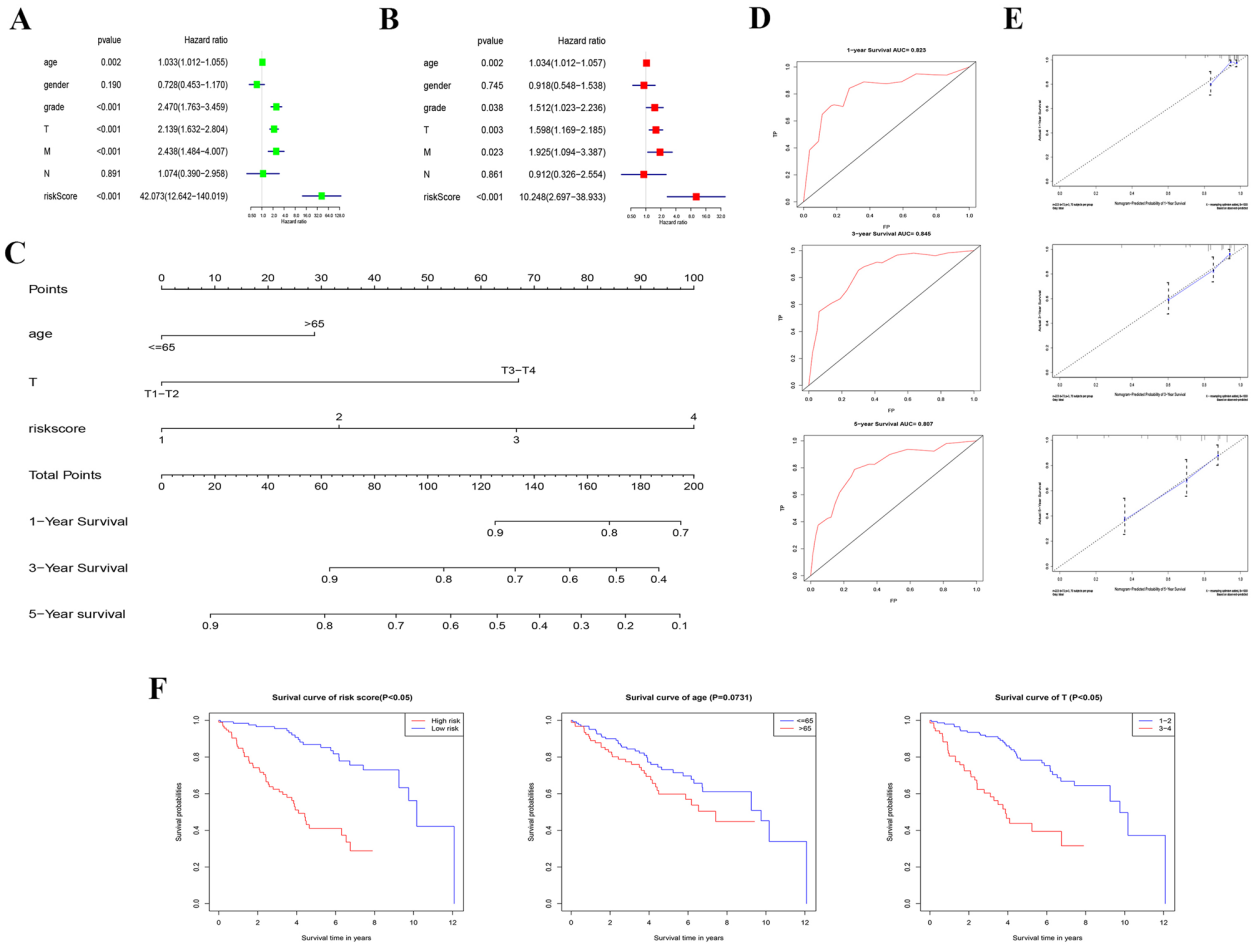
Prognostic signature of the four related regulators in the training set of independent prognostic factors for KIRC OS. (**A**,**B**) The training set of forrest plot of the independent prognostic factors in KIRC. (**C**) The nomogram of the risk model for predicting the OS probability of ccRCC patients. The whole points projected on the bottom scales indicate the likelihood of 1-, 3-, and 5-year OS. (**D**) AUC of the ROC analysis showed the predicted efficacy of the risk model in the training set. (**E**) The calibration plot for the nomogram predicting 1-, 3-, and 5-year OS. The y-axis indicates the actual survival, as measured by the K–M analysis, while the x-axis shows the nomogram-predicted survival. (**F**) Kaplan–Meier survival curves stratified according to clinicopathological and risk scores.

The independent prognostic factors included age, grade, M, and risk score through univariate and multivariate analysis (p < 0.05) (Fig. 4A,B). Furthermore, we take P < 0.01 as the standard to include age, T, and riskscore in Norman Graph (Fig. 4C). AUC of 1-, 3-, and 5-year ROC curve is 0.823, 0.845 and 0.807, respectively (Fig. 4D) and the C-index was 0.764 (Table S3). The Norman Chart model has better prediction ability verified by calibration curve. (Fig. 4E). The survival curve of risk score shows that low-risk group has better prognosis than high-risk group. The patients of T1-T2 were better than the patients of T3-T4 to survive rate (Fig. 4F).

### Effects of m1A regulatory factor modifications on immune microenvironment

**Figure 5:**
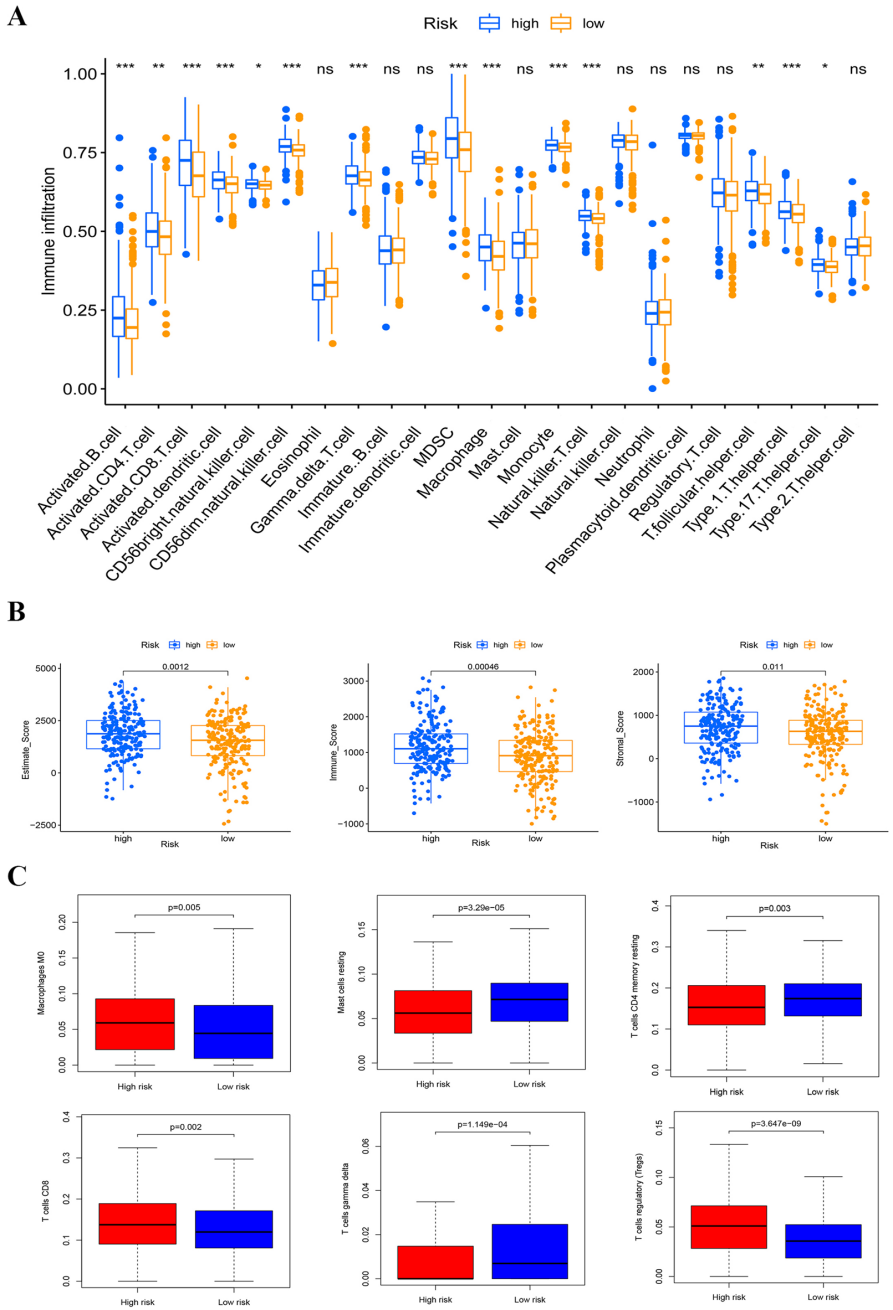
TME cell infiltration characteristics in distinct modification patterns. (**A**) Box plot for the TME cells in distinct risk groups derived from KIRC patients based on the ssGSEA. The asterisks represented the statistical p value (*p < 0.05; **p < 0.01; ***p < 0.001). (**B**) Immune, stromal and ESTIMATE scores within the low- and high-risk groups. (**C**) Differential analysis of immune cells in two low-risk groups.

**Figure 6:**
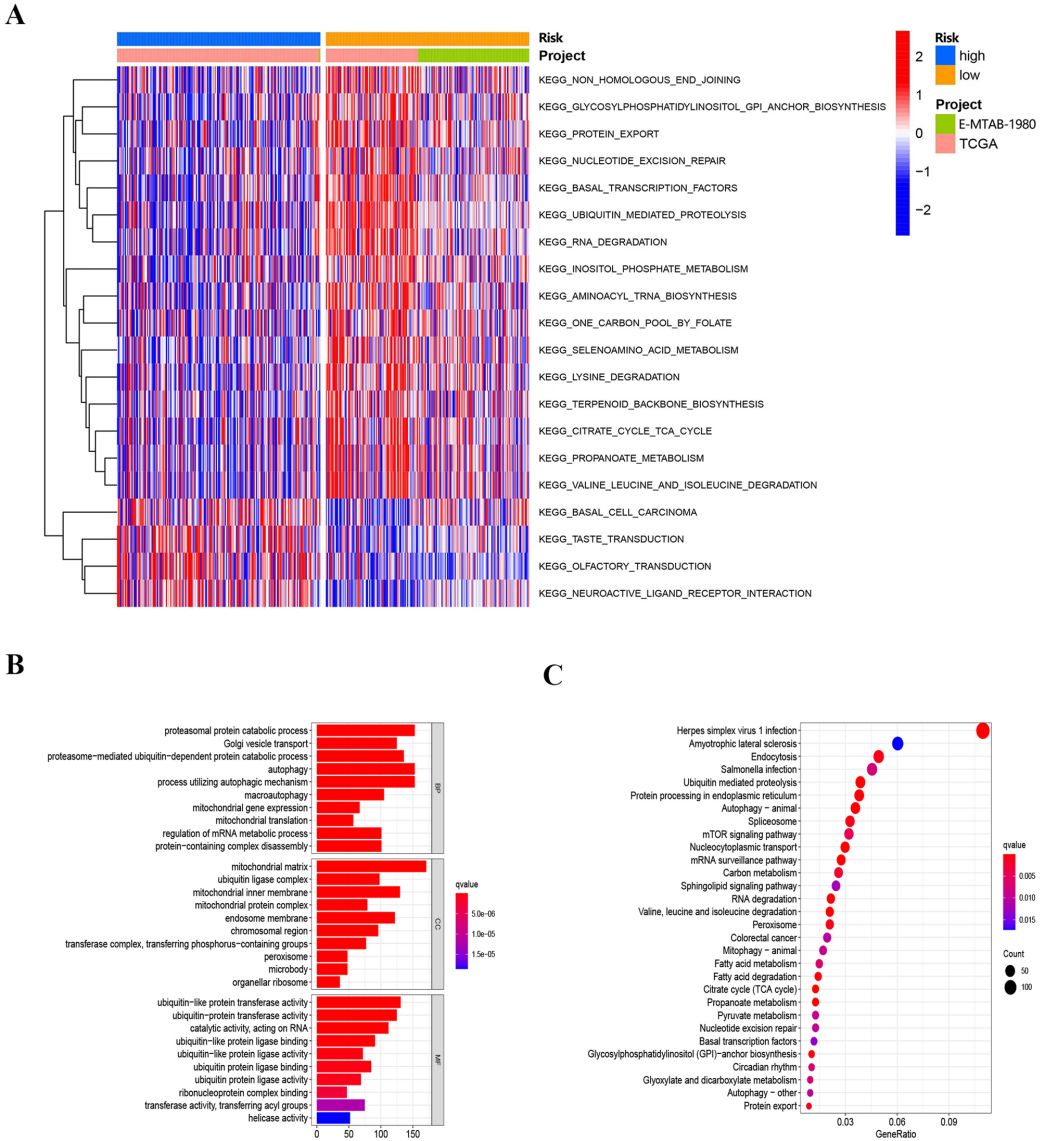
Biological characteristics of m1A phenotype–related genes. (**A**) The activation states of biological pathways between low- and high-risk groups using GSVA enrichment analysis. (**B**) Functional annotation for overlapping m1A phenotype–related genes using GO enrichment analysis. (**C**) Functional annotation for overlapping m1A phenotype–related genes using and KEGG enrichment analysis.

Nowadays, more and more tumor pathogenesis point to tumor immune microenvironment. Therefore, it is necessary to conduct ssGSEA analysis for assessing the correlation between immune infiltrating cells and risk. In the low-risk group, the abundance of most immune cells is significantly lower than that the high-group. Especially activated B cells, activated CD8 T cells, myeloid-derived suppressor cells (MDSCs), and Macrophage etc. (Fig. 5A). Then, we studied the correlation between risk and tumor microenvironment in ccRCC. In the low-risk group, the immune, stromal, and ESTIMATE scores were significantly lower than that the high-group by the results of immune cell infiltration analysis (Fig. 5B). The proportion of infiltrated immune cells to CIBERSORT algorithm in ccRCC, we found that there was a significant difference in the enrichment of immune cells between the low–high risk groups. Macrophages M0, mast cells resting, T cells CD4 memory resetting, T cells gamma delta, T cells CD8 and regulatory T cells (Tregs) were significantly enriched in low–high risk groups. In the high-risk group, the enrichment of Macrophages M0, T cells CD8 and T cells regulatory (Tregs) is more obvious than that in the low-risk group. Mast cells resting, T cells CD4 memory resting and T cells gamma delta are more enriched in low-risk groups (Fig. 5C). These results highlight the differences in immune cell types between two group populations. Therefore, the study of immune cell infiltration in ccRCC based on m1A regulatory gene may help clarify its mechanism and improve prognosis prediction. GSVA enrichment analysis results show that high group was enriched in basal cell carcinoma, taste transduction, olfactory transduction, and neuroactive ligand receptor interaction. And low group was enriched in ubiquitin mediated proteolysis, RNA degradation, protein export, aminoacyl TRNA biosynthesis, and selenite metabolism etc. (Fig. 6A).

### GO and KEGG analysis in risk score

In two risk groups, a total of 2997 m1A phenotype-related differentially expressed genes (DEGs) were screened out. The result in Fig. 6B,C, based on KEGG analysis, DEG has biological functions in Protein processing in endothelial reticulum^26^, Spliceosome^27^, mTOR signaling pathway^28^, and colorectal cancer, as well as in proteomic protein catalytic process, autophagy^29^, regulation of mRNA metabolic process^30^, ubiquitin − like protein^31^ and ubiquitin protein^32^ based on GO analysis. The enrichment results showed that DEGs were significantly related to tumor progression and immune responses.

### Immune checkpoints related to the risk groups

**Figure 7:**
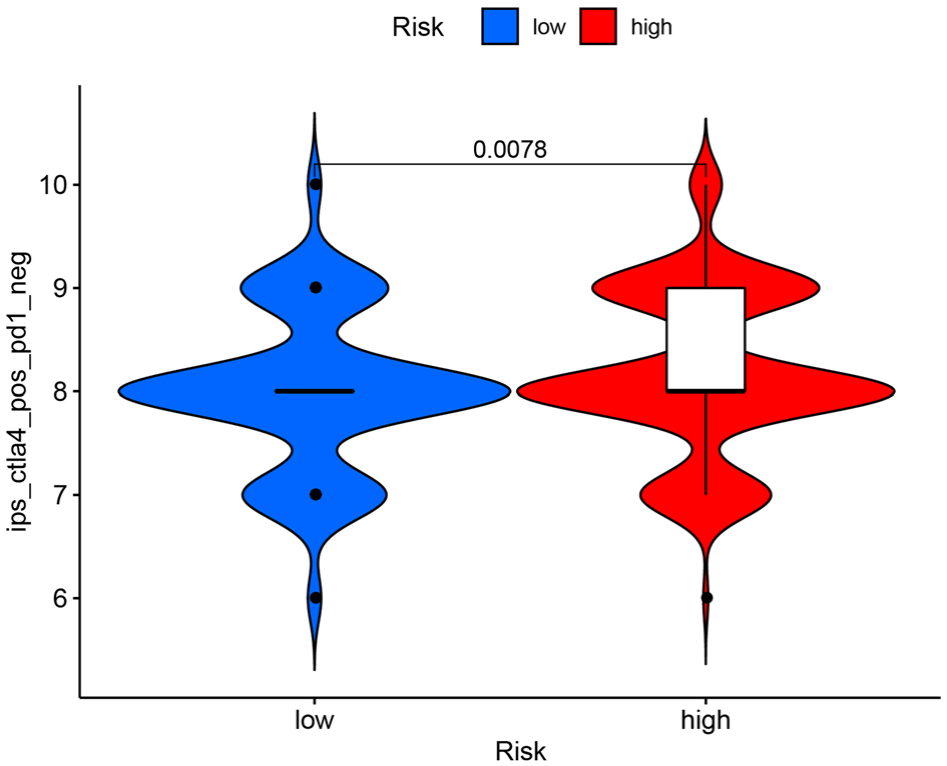
High-risk group was more likely to respond to anti-CTLA4 immunotherapy. *ICPs* immune checkpoints, *ICBs* immune checkpoint inhibitors.

The latest research shows that the treatment of immunocheckpoint inhibitors (ICIs) is changing the therapeutic prospects of urological tumors^33^. Therefore, based on the data samples of ccRCC, cytotoxic T lymphocyte associated protein 4 (CTLA-4) and programmed cell death protein 1 (PD-1) which were current mainstream immunocheckpoint molecular in tumors were explored. In our study, the high-risk is relative to the low-risk, the immunotherapy score was higher. It’s meaning anti-CTLA-4 treatment is useful to the high-risk group patient (Fig. 7).

### Screening potential drugs for two groups

**Figure 8:**
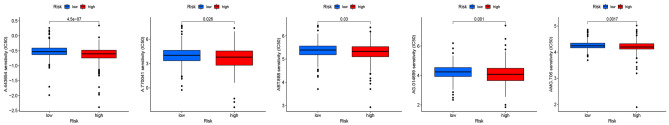
Potential therapeutic drugs related to ccRCC of five potential drugs.

The IC50 of each sample was estimated, and the IC50 of five drugs was different between the two risk groups. We have obtained 5 potential drugs for ccRCC (A.443654, A.770041, ABT.888, AG.014699, AMG.706) and their molecular structures in low–high risk groups (Fig. 8). The five drugs are more sensitive in high-risk group. These drugs may play an important role in the treatment of ccRCC and provide more drug options for clinic, but the effectiveness and practicability need further research for ccRCC.

### Analysis of interacting proteins linked to four prognostic genes

**Figure 9:**
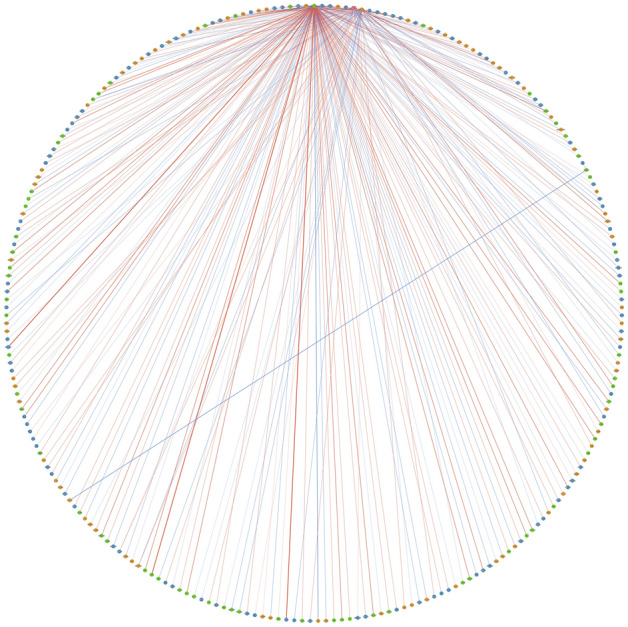
Combining YTHDF1, TRMT61B, TRMT10C and ALKBH1 to construct protein interaction network in ccRCC.

**Figure 10:**
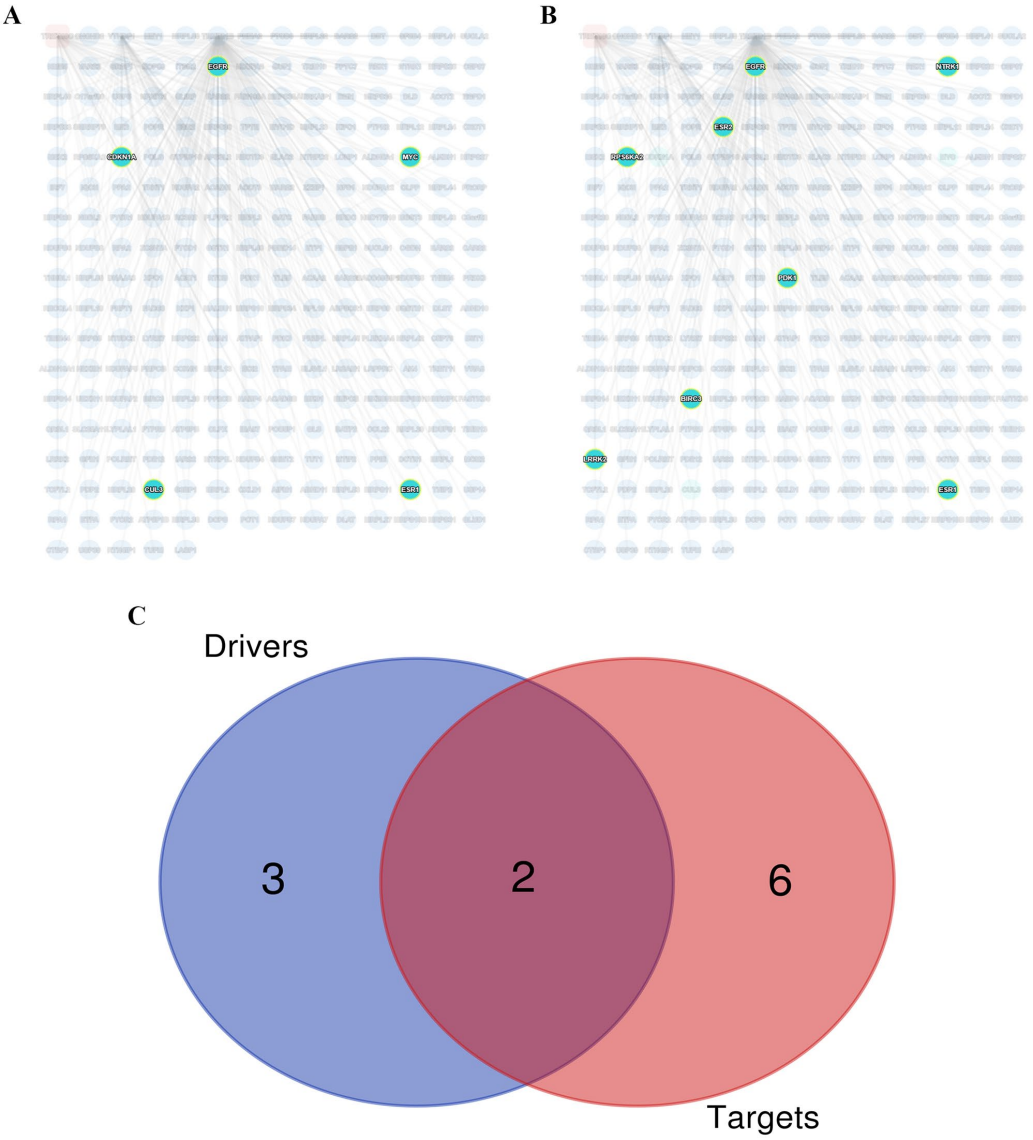
Interacting proteins Linked to Four Prognostic Genes. (**A**) Tumor drivers in interactive networks. (**B**) Drug targets in interactive networks. (**C**) An intersection analysis of tumor drivers and drug targets was conducted.

**Figure 11:**
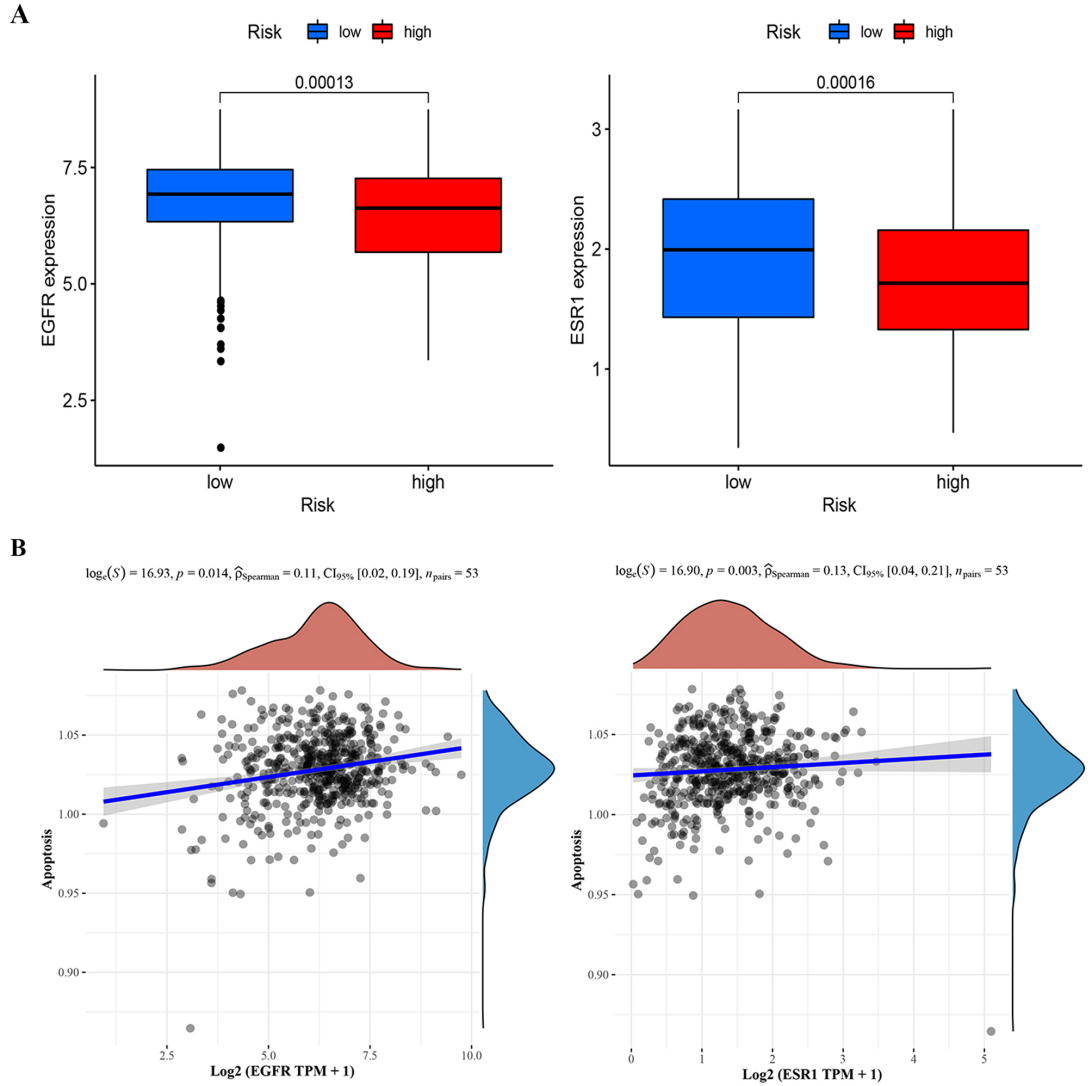
Analysis of interacting proteins linked to four prognostic genes. (**A**) Expression of EGFR and ESR1 in high and low risk groups. (**B**) EGFR and ESR1 are related to apoptosis.

Four prognostic genes were included in the network and 243 related genes were obtained. The relationship between them is shown in the figure (Fig. 9). Next, we filter out 8 cancer drivers and 5 drug targets (Fig. 10A,B). Display the intersection result with Venn chart (Fig. 10C). EGFR and ESR1 are confirmed as tumor drivers and drug targeting factors in ccRCC. Then we observed whether the EGFR and ESR1 were different between the two groups. The results proved that the expression of EGFR and ESR1 have differences in two risk groups (Fig. 11A). The results of pathway correlation revealed that both were positively correlated with apoptosis (Fig. 11B).

### Validation of the risk signature

**Figure 12:**
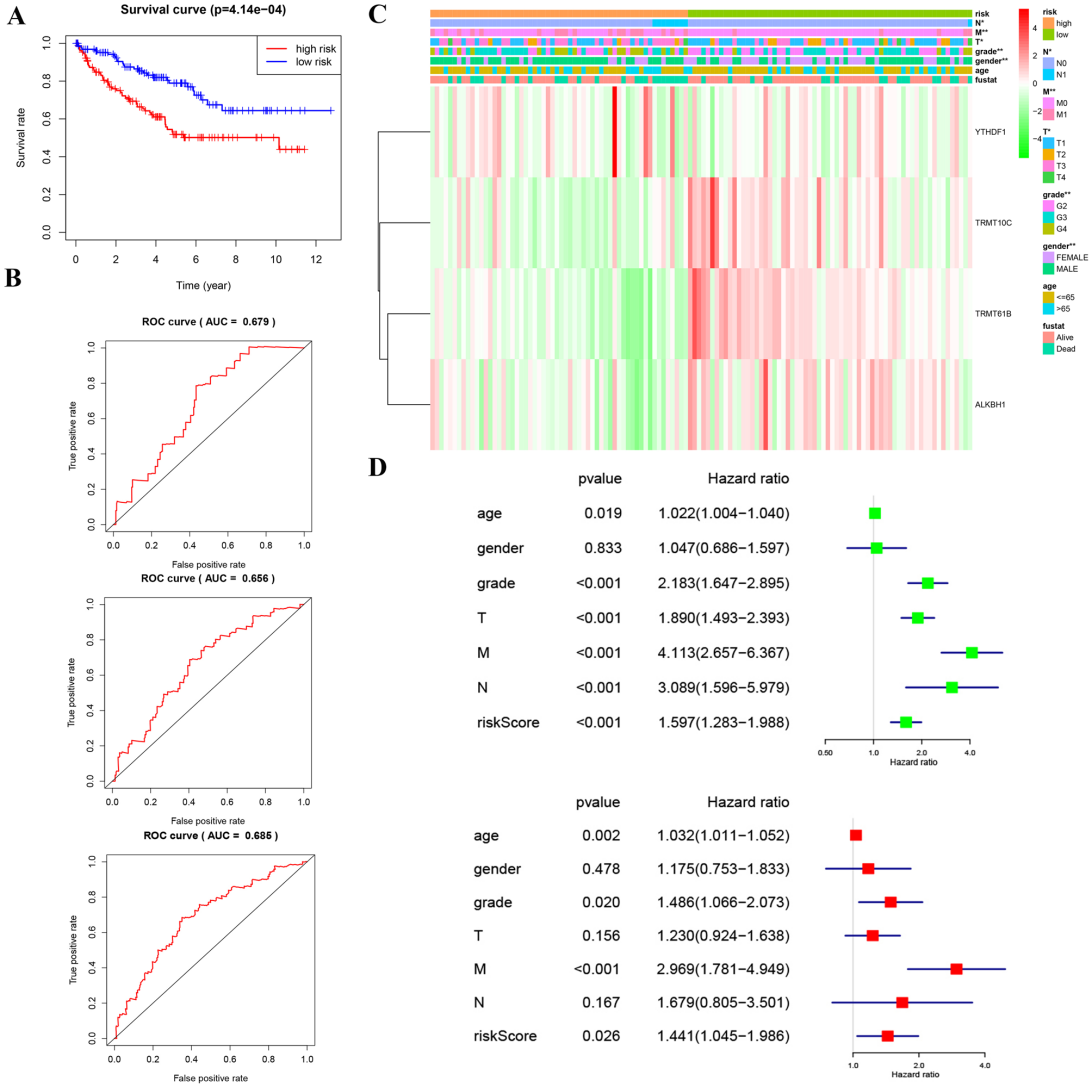
Verification of m1A Gene Signatures in ccRCC. (**A**) Kaplan–Meier survival curves for OS in the two groups of low and high risk. This analysis was based on the survival information of samples in the validation set. (**B**) The validation set of the ROC curve for evaluating the prediction efficiency of the prognostic signature. (**C**) The validation set of the heat map of the relationship between the gene expression of the corresponding four regulatory factors and clinical features. (**D**) The validation set of the forest plot of the independent prognostic factors in KIRC.

The validation set (n = 265) is used to validate the prognostic risk characteristics we have constructed. Kaplan Meier curve showed that the prognosis of low-risk group was better (p < 0.05) (Fig. 12A). 1-, 3-, and 5-year ROC curve also proves that risk score has good prediction ability (Fig. 12B). AUC is 0.679, 0.656 and 0.685 respectively. The prognostic model results are highly consistent with the training set results. (Fig. 12C). Consistent with the training set, univariate analysis demonstrated that age, grade, T, and M significantly associated with the risk in validation set. Risk score was remained to an independent prognostic factor by multivariate analysis in validation set (Fig. 12D). The above results basically confirmed that the prognostic risk model we built is reliable.

### The expression of four genes in ccRCC and apoptosis rate of two genes in ccRCC

**Figure 13:**
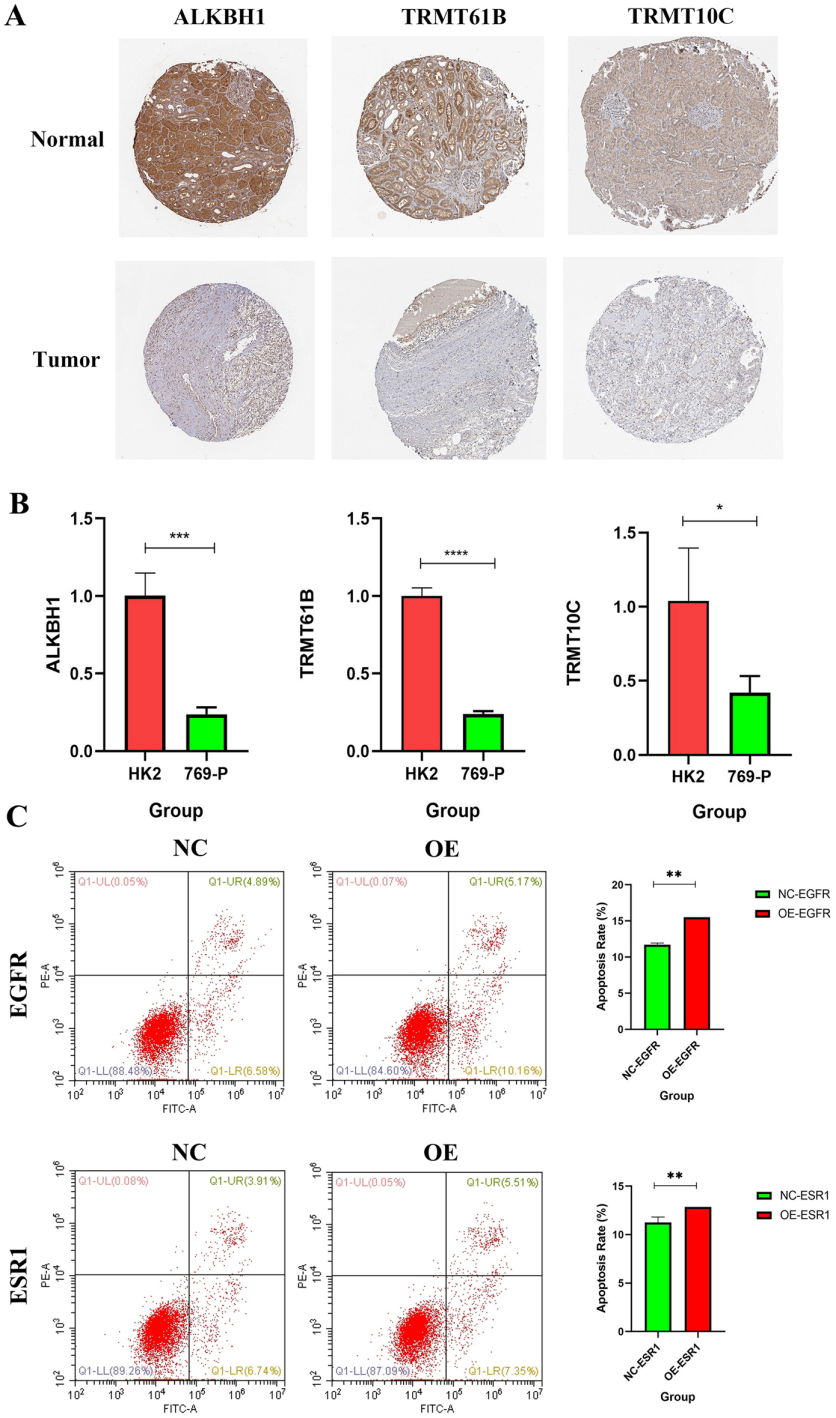
Expression level of YTHDF1, TRMT61B, TRMT10C and ALKBH1 in clear cell renal cell carcinoma (KIRC). (**A**)Protein expression of TRMT61B, TRMT10C and ALKBH1 in KIRC. KIRC, kidney renal clear cell carcinoma. (**B**)The mRNA expression of TRMT61B, TRMT10C and ALKBH1 in ccRCC cell lines (HK2, 769-P) was detected by the qRT-PCR assay. *p < 0.05, **p < 0.01, ***p < 0.001, and ****p < 0.0001. (**C**) After knockdown of EGFR and ESR1, cell apoptosis in each group was detected by flow cytometry.

According to available literature reports, low expression of YTHDF1 in ccRCC tissues^34^. We use HK2 (epithelial cells;) and 769-P (ccRCC) cell lines to verify the expression of other three genes in ccRCC. The expression of TRMT61B, TRMT10C and ALKBH1 in 769 cell line is lower than that in HK2 cell line (Fig. 13A). The results of immunohistochemistry support our conclusion. The staining intensity of TRMT61B, TRMT10C and ALKBH1 in tumor tissue is higher than that in normal kidney tissue (Fig. 13B). The results of flow apoptosis were consistent with our conclusions of bioinformatics. Both are positively correlated with apoptosis, and are protect factors. After overexpression of EGFR and ESR1, the apoptosis rate of OE group decreased compared with the NC group (Fig. 13C).

## Discussion

More and more evidence proved that m1A regulatory genes participate widely in the mechanism of many diseases^35^. Nowadays, the research of tumor immunity and tumor targeted drugs play a key role in the treatment and prognosis of tumors^36,37^. Based on the 10 m1A regulators, we determined the risk label of four genes YTHDF1, TRMT61B, TRMT10C and ALKBH1. The new prognosis model can more accurately predict the OS of patients with ccRCC, and the reliability of the model has been verified. Most immune cells are infiltrated in the low-risk group. The immune, stromal and ESTIMATE scores were significantly abundant in the high-risk group by tumor immune infiltration analysis. Interestingly, this result is contrary to the conclusion that the enrichment of immune infiltrating cells in some tumors indicates a good prognosis^38,39^. However, it has been clarified that the immune system can both inhibit tumor growth and promote tumor development by changing tumor immunogenicity or promoting immunosuppressive state^40^. So, we have reason to think that TME may be a double-edged sword. ccRCC are characterized by abundant leukocyte infiltration, usually including CD8 + T cells, CD4 + T cells, and macrophages^41,42^. Many CD8 + and CD4 + T cells in the tumor are associated with poor prognosis^40^. In addition, type 1 T helper cells participate in cell-mediated immune response, so type 2 T helper cells reaction tends to contribute to the immunosuppressive state of RCC tumors. type 2 T helper cells promote inflammatory tumor microenvironment and enhance the tumor promoting function of myeloid cells^43,44^. In addition, the level of MDSCs in patients with ccRCC increased and was positively correlated with poor prognosis^45–48^. However, the complexity of RCC tumor TME still exists^49–51^. For example, the results of NK cells are different from our conclusions. In this study, NK cell infiltration in the high-risk group was more than that in the low-risk group. But some studies show that a higher proportion of NK cells in tumour-infiltrating lymphocytes (TILs) is associated with better prognosis in RCC^52–54^. The results of CIBERSORT further clarified the differences in the expression of several immune cells in the two groups. Enrichment of Macrophages M0^55^, T cells CD8 (Intratumor CD8 + T-cell infiltration indicates poor clinical prognosis for ccRCC patients)^56^ and Tregs (Tregs were associated with adverse clinical outcomes in RCC)^57^ means poor prognosis. High expression of mast cells resting, T cells CD4 memory resting and T cells gamma delta in low-risk group corresponds to good prognosis. KCNN4 is significantly negatively correlated with mast cell resting, and the high expression of KCNN4 may lead to greater possibility of immune escape^58^. Similarly, the proportion of immune cell infiltration in low-risk group of T cells CD4 memory resting is higher than that in high-risk group in colorectal cancer^59^. γδ T cells have a recognized protective role in cancer, mainly based on their strong cytotoxicity and interferon- γ Generation of^60^. In addition, GSVA enrichment analysis revealed that the high-risk group was related to the mechanisms of basal cell carcinoma, taste conduction, olfactory conduction, and the interaction of neuroactive ligand receptors. Therefore, we think that the m1A risk model may affect the TME in ccRCC, which may affect the prognosis of ccRCC. CTLA-4, as an oncogene, accelerates the development of ccRCC and has high prognostic value. The results of our immune checkpoint showed that the high-risk group had good immunotherapy effect against CTLA-4^61^. The most common tumor in the urinary system is RCC^62^. It is insensitive to radiotherapy and chemotherapy. Therefore, it is necessary to screen small molecule targeted drugs for RCC^62^. In our results, A.443654, A 770,041, ABT.888, AG.014699 and AMG.706 are more sensitive in high-risk group. Akt and mTOR are therapeutic targets for cancer treatment, and A.443654 is an AKT inhibitor. Zheng et al. found that A.443654 may have a potential positive effect on the treatment of breast cancer^63^. ABCB1/Pgp mediated chemoresistance can be reversed by small molecule inhibitor A-770041 And it has clinical effect on multidrug resistant osteosarcoma^64^. ABT.888 is a PARP inhibitor, also called Veliparib^65^. Some studies have proved that ABT.888 can promote the apoptosis of drug resistant melanoma cells by inhibiting the migration and invasion of melanoma cells^66^. AG.014699, Rucaparib, is PARP inhibitor^65^. Clinical research shows that Rucaparib has antitumor activity in patients with metastatic castration-resistant prostate cancer and a deleterious BRCA alteration^67^. AMG 706 is an endothelial cell proliferation inhibitor, considered as a potential agonist of MrgprF, which can inhibit tumor growth in vivo and in vitro^68^. AMG 706 can inhibit tumor cell proliferation and migration, and promote apoptosis in cutaneous melanoma by reducing Akt phosphorylation and PI3K/Akt signaling^68^. We further analyzed the four genes of the model. EGFR and ESR1 are obtained from the intersection of tumor drivers and tumor targeting drug factors in the interaction network. The expression of the two genes was significantly different. The both genes were highly expressed in the low-risk group. In addition, the following pathway correlation analysis found that EGFR and ESR1 were positive correlated with tumor cell apoptosis, which further supported our conclusion. In addition, the model established by Chen et al. based on the m6A methylation regulatory factor and the model established by Li et al. based on the m5C methylation regulatory factor were both subjected to single ROC validation^69,70^. However, our model is based on the m1A adjustment factor, and the area under the 1-, 3-, and 5-year receiver operating characteristic of the model shows that the model is reliable. This result was also validated in the external validation set.

The construction of our model has been verified by the validation set, but our research still has shortcomings. First, we need experiments and clinical trials to verify our results; Secondly, our research is based on the m1A regulator, without in-depth study of the mechanism of methylation. In addition, the research on EGFR and ESR1 is not deep enough.

### Supplementary Information

Supplementary Information.

Supplementary Legends.

## Data Availability

The data used in this study was obtained through TCGA (https://portal.gdc.cancer.gov/) and ArrayExpress (https://www.ebi.ac.uk/arrayexpress) online databases.
