# Supplementary material for: Predicting response of immunotherapy and targeted therapy and prognosis characteristics for renal clear cell carcinoma based on m1A methylation regulators
Source: Sci Rep. 2023 Aug 4;13:12645. doi: 10.1038/s41598-023-39935-4 (PMC10403615; doi:10.1038/s41598-023-39935-4)
Supplement: Supplementary file 2 — Supplementary Legends. [file 41598_2023_39935_MOESM2_ESM.docx]

Supplementary information

Supplementary Table 1 | The LASSO regression analysis results of four prognostic factors.

Supplementary Table 2 | Kaplan Meier analysis results on four genes.

Supplementary Table 3 | C index of model validation.
